# Integrative analysis of intestinal flora and untargeted metabolomics in attention-deficit/hyperactivity disorder

**DOI:** 10.3389/fmicb.2025.1452423

**Published:** 2025-01-29

**Authors:** Jiamin Lu, Maoying Jiang, Dingyue Chai, Yuzi Sun, Lihui Wu

**Affiliations:** ^1^Departments of Basic Medicine and Forensic Medicine, Hangzhou Medical College, Hangzhou, China; ^2^Behavioral Pediatric Department and Child Primary Care Department, Hangzhou Children’s Hospital, Hangzhou, China

**Keywords:** attention deficit hyperactivity disorder, intestinal flora, metagenomics, metabolomics, fecal microbial transplantation

## Abstract

Attention Deficit Hyperactivity Disorder (ADHD) is a clinically common neurodevelopmental disorder of the brain. In addition to genetic factors, an imbalance in gut flora may also play a role in the development of ADHD. Currently, it is critical to investigate the function of gut flora and related metabolites, which may form the fundamental basis of bidirectional cross-linking between the brain and the gut, in addition to focusing on the changed gut flora in ADHD. This study aimed to investigate the possible relationship between changes in gut flora and metabolites and ADHD by analyzing metagenome and untargeted metabolomics of fecal samples from ADHD patients. Specifically, we attempted to identify key metabolites and the metabolic pathways they are involved in, as well as analyze in detail the structure and composition of the gut flora of ADHD patients. In order to further investigate the relationship between gut flora and ADHD symptoms, some behavioral studies were conducted following the transplantation of gut flora from ADHD patients into rats. The results of the metagenome analysis revealed several distinct strains, including *Bacteroides cellulosilyticus*, which could be important for diagnosing ADHD. Additionally, the ADHD group showed modifications in several metabolic pathways and metabolites, including the nicotinamide and nicotinic acid metabolic pathways and the metabolite nicotinamide in this pathway. The behavioral results demonstrated that rats with ADHD gut flora transplants displayed increased locomotor activity and interest, indicating that the onset of behaviors such as ADHD could be facilitated by the flora associated with ADHD. This research verified the alterations in gut flora and metabolism observed in ADHD patients and provided a list of metabolites and flora that were significantly altered in ADHD. Simultaneously, our findings revealed that modifications to the microbiome could potentially trigger behavioral changes in animals, providing an experimental basis for comprehending the function and influence of gut flora on ADHD. These results might provide new perspectives for the development of novel treatment strategies.

## 1 Introduction

The gut is not only a key component of the digestive system but also a crucial component of the immune system. An independent neurological system called the enteric nervous system exists in the gastrointestinal tract. Millions of neurons make up the enteric nervous system, which is closely connected to the brain and controls the activity, secretory function, and blood circulation of the gut (Dabke et al., 2019; Margolis et al., 2021). Gut microbiome refers to the vast population of bacteria that reside in the human gut (Backhed et al., 2005). The microbiota create a complex ecosystem that is essential for controlling metabolism in addition to aiding in food digestion, detoxifying medications and poisons, and maintaining the health of the immune system (Farzi et al., 2018; Frisbee and Petri, 2020; Zeineldin et al., 2018). There may be a significant metabolic potential for the bacteria in the gut microbiota as their number is similar to that of human cells (Sender et al., 2016). In addition, gut microbes can reduce inflammation, modify gene expression, and affect the synthesis of neurotransmitters - particularly the latter as they have a direct bearing on behavior and mood (Sasso et al., 2023). Clinical problems such as obesity, diabetes, cardiovascular disease, and allergies are significantly linked to an imbalance in the gut microbiota (Fukuda et al., 2011; Kelly et al., 2016; Orivuori et al., 2015; Safari et al., 2019). Furthermore, gut microbial dysbiosis has been linked to several types of neurological conditions, including attention deficit/hyperactivity disorder (ADHD), depression, anxiety disorders, and autism spectrum disorders (Aizawa et al., 2016; Bundgaard-Nielsen et al., 2020; Cheng et al., 2020; Kang et al., 2013; Reimherr et al., 2017; Rosenfeld, 2015; Sharon et al., 2019). Research has indicated that altering the composition of the gut microbiota may be a useful strategy for both treating and preventing certain neurological conditions. Thus, designing novel therapeutic approaches requires a thorough understanding of the gut microbiota and its roles.

ADHD, a highly heritable neurodevelopmental disorder, is characterized by distractibility, hyperactivity, and impulsivity (Martinez-Badia and Martinez-Raga, 2015; Wustner et al., 2019). Patients with ADHD experience profound consequences in social and emotional management as well as in their academic and professional lives (Usami, 2016). Numerous elements, including pathophysiology, neurobiology, etiology, and genetics, are involved in the complicated pathogenesis of ADHD (Hinshaw, 2018). There is currently no solid evidence regarding the precise causes of ADHD. According to recent research, changes in gut flora may be linked to the onset of ADHD.

Three main pathways explain the relationship between gut microbiota and ADHD: the microbes directly produce metabolites and neurotransmitters that affect the nervous system (Logsdon et al., 2018), they act on the vagus nerve to affect the brain (Carabotti et al., 2015), and they stimulate peripheral immune cells to affect the blood–brain barrier and other nervous system factors (Amini-Khoei et al., 2019; Fung et al., 2017). There is a notable variation in the intestinal flora composition of ADHD patients when compared to the healthy population, according to an investigation of their gut flora (Wan et al., 2020). The overall composition and relative abundance of the flora reflect this variation; however, there is currently inconclusive research in this area. Focusing on particular microbiota, it was discovered that Bifidobacterium, Odorobacterium, Enterococcus, Neisseria, and Clostridium showed an increasing trend in relative abundance when studied in ADHD, while Microbacterium, Lachnoclostridium, Faecalibacterium, and Parabacterium showed a decreasing trend in relative abundance (Ming et al., 2018; Wang et al., 2020). Szopinska-Tokov showed that the Ruminococcaceae_UGC_004 and inattentive symptoms of ADHD are related (Szopinska-Tokov et al., 2020). This underscores the possibility that alterations in gut flora are linked to the neuropathological mechanisms of ADHD. These findings add to our knowledge of the pathogenic mechanisms underlying ADHD and may provide key hints for the creation of novel therapeutic strategies. Nevertheless, more thorough research is required to gain a more precise understanding of the connection between gut flora and ADHD.

While the interaction between the brain and gut microorganisms is a topic of current research, a complete understanding of the mechanism involved may not always be revealed by concentrating only on the gut germs. As the foundation for two-way communication between the brain and gut flora, attention must also be given to the impact of gut flora on metabolite production. According to some research, the brain-gut axis affects the brain by regulating the generation and function of neurotransmitters via vagal stimulation, immune system reactions, bacterial metabolites, or activation of the hypothalamic–pituitary–adrenal (HPA) axis (Bonaz et al., 2019; De Punder and Pruimboom, 2015; Eicher and Mohajeri, 2022; Fu et al., 2023). Simultaneously, the brain can modify gut secretion, peristalsis, and sensation, which in turn can affect the makeup and activity of the gut bacteria (Margolis et al., 2021). In the case of ADHD, for instance, an increase in certain gut microorganisms such as Bifidobacterium may raise blood tryptophan levels (Desbonnet et al., 2008). This can then be converted to 5-hydroxytryptophan in the brain, which has an impact on mood and cognitive performance. Additionally, phenylalanine, a precursor of the neurotransmitters dopamine and norepinephrine (Aarts et al., 2017), which are essential for mood regulation and cognitive function, is increased by Bifidobacterium (Coleman et al., 2019). Disturbances in these neurotransmitters may be linked to pathomechanisms related to ADHD. Thus, variations in the gut microbiota’s composition may result in variations in neurotransmitter production, which may then play a role in the emergence of neurological diseases like ADHD. The aforementioned results highlight the significance of gaining a more comprehensive understanding of the function of gut flora and metabolites in disease preventive and treatment approaches.

In summary, the properties of the gut microbiota and metabolites in ADHD, as well as their interactions, are not well understood. The relationship between alterations in gut microbiota and metabolites and ADHD has not been clearly established by previous research. In order to detect specific alterations in microbiota and metabolites in the ADHD gut and investigate the relationship between them, our study evaluated shotgun metagenomic and untargeted metabolomics from ADHD patients and healthy controls in tandem. To investigate the relationship between gut microbiota and symptoms of ADHD, we also transplanted microbiota from ADHD patients into Sprague–Dawley (SD) rats and assessed the impact these microbiota produced in the rats. We hope to provide new perspectives for the development of novel ADHD treatment plans.

## 2 Methods

### 2.1 Participant selection

During July and August of 2020, five children who had been initially diagnosed with ADHD were chosen from Hangzhou Children’s Hospital. The following criteria had to be met for a subject to be included: (1) participants had to meet the DSM-V criteria for ADHD diagnosis (Austerman, 2015; Posner et al., 2020); (2) participants could not have serious organic diseases affecting the neurological, hepatic, cardiovascular, or hematopoietic systems; (3) participants could not have a history of digestive or other chronic diseases; (4) participants could not have an allergy such as allergic rhinitis or asthma; (5) participants’ body mass index could not be less than 20 kg/m2; and (6) participants had to be free of probiotics, related traditional Chinese medicine, psychostimulant drugs, etc. and other combined treatment methods such as electroencephalography biofeedback. The healthy control (HC) group consisted of eight children from various households who were chosen at the same time and under the same inclusion criteria, with the exception that the HCs did not have an ADHD diagnosis based on the DSM-V criteria. The Conners Parent Rating Scale was utilized by the parents of each participant involved to evaluate the degree of symptoms associated with ADHD. Feces samples were obtained between 8:00 and 12:00 a.m. Before testing, the stool samples were transported at 4°C and stored at −80°C. The parents or legal guardians of the subjects provided verbal informed consent.

### 2.2 Shotgun metagenome sequencing of fecal samples

DNA was extracted from human feces samples using the hexadecyl trimethyl ammonium bromide method. DNA degradation degree, potential contamination and DNA concentration were measured using an Agilent 5,400. Sequencing libraries were generated using the NEBNext@ UltraTM DNA Library Prep Kit for Illumina. The library preparations were sequenced on an Illumina Novaseq 6,000 platform and paired-end reads were generated. The raw data of bacteria, fungi and viruses in human feces samples were obtained by metagenomic sequencing using the Illumina Novaseq high-throughput sequencing platform. In order to ensure the reliability of data, the raw sequencing data was preprocessed using Kneaddata software. Kraken2 and the self-build microbial database were used to identify the species contained in the samples, and then Bracken was used to predict the actual relative abundance of species in the samples. The clean reads after quality control and de-host were used to obtain a blast database (UniRef90) using HUMAnN2 software, and the annotation information and relative abundance table from each functional database were obtained according to the corresponding relationship between Uniref90 ID and each database. Based on the species abundance table and functional abundance table, abundance clustering analysis, Principal Coordinates Analysis (PCoA) analysis (species only) and sample clustering analysis were performed. When grouping information was available, Linear discriminant analysis Effect Size (LEfSe) Biomarker and Dunn test analyses were performed to excavate differences in species composition and functional composition between samples.

### 2.3 Untargeted metabolomics of fecal samples

Analysis was performed using an ultra-high performance liquid chromatography coupled to a quadrupole time-of-flight (TOF) mass spectrometer. For hydrophilic interaction chromatography separation, samples were analyzed using a 2.1 mm × 100 mm ACQUIY UPLC BEH Amide 1.7 μm column. In both ESI positive and negative modes, the mobile phase contained A = 25 mM ammonium acetate and 25 mM ammonium hydroxide in water and B = acetonitrile. The gradient was 95% B for 0.5 min and was linearly reduced to 65% in 6.5 min, and then reduced to 40% in 1 min and kept for 1 min, and then increased to 95% in 0.1 min, with a 3 min re-equilibration period employed. The ESI source conditions were set as follows: Ion Source Gas1 (Gas1) as 60, Ion Source Gas2 (Gas2) as 60, curtain gas (CUR) as 30, source temperature: 600°C, IonSpray Voltage Floating (ISVF) ± 5,500 V. In mass spectrometry (MS) only acquisition, the instrument was set to acquire over the m/z range 60–1,000 Da, and the accumulation time for the TOF MS scan was set at 0.20 s/spectra. In auto MS/MS acquisition, the instrument was set to acquire over the m/z range 25–1,000 Da, and the accumulation time for product ion scan was set at 0.05 s/spectra. The product ion scan was acquired using information dependent acquisition (IDA) with high sensitivity mode selected. The parameters were set as follows: the collision energy (CE) was fixed at 35 V with ±15 eV; declustering potential (DP), 60 V (+) and − 60 V (−); exclude isotopes within 4 Da, candidate ions to monitor per cycle: 10. The raw MS data were converted to MzXML files using ProteoWizard MSConvert. For peak picking, the following parameters were used: centWave m/z = 10 ppm, peakwidth = c (10, 60), prefilter = c (10, 100). For peak grouping, bw = 5, mzwid = 0.025, and minfrac = 0.5 were used. CAMERA (Collection of Algorithms of Metabolite pRofile Annotation) was used for annotation of isotopes and adducts. In the extracted ion features, only the variables having more than 50% of the nonzero measurement values in at least one group were kept. Compound identification of metabolites was performed by comparing the accuracy of m/z value (<10 ppm), and MS/MS spectra with an in-house database established with available authentic standards.

### 2.4 Fecal microbial transplantation and behavioral experiments

#### 2.4.1 Animals

Male SD rats were purchased from Beijing Viton Lihua Laboratory Animal Co. All the rats were given free access to food and water and were gradually acclimated to the laboratory conditions (12-h light/dark cycle, 22 ± 1°C, and 55 ± 5% relative humidity). To reduce animal suffering, the number of animals utilized in the study was kept to a minimum.

#### 2.4.2 Fecal microbial transplantation

In order to eliminate individual differences, fecal samples from five ADHD patients and eight HCs were processed and mixed in sterile tubes. To obtain the final bacterial solution for usage, 1 g of feces was combined with 10 mL of sterile PBS, vortexed for approximately 10 min, and then filtered through 200, 400, and 800 mesh in that order. The configured bacterial solution was stored at −80°C after adding glycerol, and the proportion of bacterial solution to glycerol was 1:1. The solution was thawed in a 37°C water bath and vortexed once more before use.

**Figure 1:**
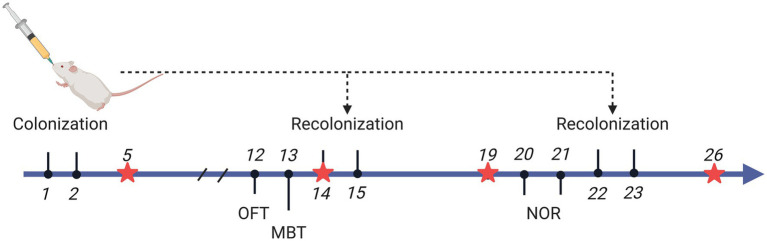
Experimental Timeline. Rats underwent microbial colonization on days 1, 2, 14, 15, 22, and 23 of the experiment, while fecal samples were collected on days 5, 14, 19, and 26. On day 14, fecal collection was performed prior to colonization. Behavioral experiments were conducted on day 12 (open field test), day 13 (marble burying test), and days 20 and 21 (training and testing for the novel object recognition test). Created with BioRender.com.

Figure 1 depicts the experiment timeframe. Four distinct animal groupings were established: Blank (no disposition), HC.T, and ADHD.T, Transoral gavage was used to populate the microbiota from HCs and ADHD patients, respectively, and sterile PBS was used to set up a sham-operated group. Rats underwent microbial colonization on days 1, 2, 14, 15, 22, and 23 of the experiment to guarantee that their gut microbiome remained consistent.

#### 2.4.3 Behavioral experiments

##### 2.4.3.1 Open field test

The experimental box for the OFT was 100 cm × 100 cm × 40 cm. The bottom surface was uniformly divided into 16 compartments, with the central area consisting of the middle four compartments and the peripheral area consisting of the remaining 12 compartments. The rats were positioned in the open field’s lower left corner at the start of the experiment, and they were allowed to wander around freely for 5 min. The system then automatically logged the data. Calculations were made to determine how long the rat spent engaging in activities in the central zone, how many times it entered the zone overall, how far it went in 5 min, and how many times it stood straight up. The total distance traveled and the activity time in the central area reflected the locomotor activity of the rats, and the number of times they stood upright reflected their curiosity and interest in exploring the outside world.

##### 2.4.3.2 Novel object recognition

The experimental equipment was a square laboratory box of 50 cm × 50 cm × 45 cm. Three objects were used: A, B, and C. Rectangles A and B were the same, while C was a colored cylinder. During the training phase, A and B were placed in the center of the experimental box. The rats were placed in the experimental box with their backs to these objects and it was ensured that the rats’ noses were equidistant from these two objects, and the rats remained in the experimental box for 10 min. In the test phase 24 h later, B was replaced by C, and the rat was placed in the same direction and distance for 5 min. Every encounter the rats had with these objects was meticulously documented, down to the number of times they made contact and the amount of time they spent moving within two to three centimeters of them. Static activities, such as maintaining a position on the object, were not regarded as exploratory behaviors; instead, actions such as patting with the paw, smelling with the nose, or licking the object with the mouth were documented as such. By comparing the frequency and duration of exploring new and old things, the cognitive ability of rats was deduced; a higher frequency and longer duration of exploring new objects indicates normal cognition. The Recognition Index (RI) was calculated as RI = Explore New Objects/ (Explore New Objects + Explore Old Objects) × 100%.

##### 2.4.3.3 Marble burying test

An identical rat cage that was used to house the rats was used as the experimental box. The experimental box was lined with padding approximately 5 cm thick. Glass marbles were gently placed on top of the padding, and were arranged neatly using the 4 × 6 rule. The rats were carefully positioned in a corner of the cage and did not touch the glass marbles when the experiment began. The observer left the test room after placement. The rats were allowed to move freely in the cage without disturbance for 30 min, during which time they were not given water or food. Thirty minutes later, the rats were taken out of the cage, taking care not to move the glass marbles. The number of marbles buried by the rats (marbles with 1/2 or more of their surface area covered were considered buried). The number of buried marbles reflected the impulsiveness of the rats, with more buried marbles indicating greater impulsiveness.

### 2.5 Statistical analyses

All sequencing data were analyzed at the Wekemo Bioincloud online site. The concept of amplicon was borrowed for intergroup diversity analysis of the metagenome. PCoA analysis based on Bray Curtis distance was performed to test the similarity of bacterial community structure between samples. Intergroup LEfSe analysis was performed to determine biomarkers from phylum to species level (LDA > 2, *p* < 0.05). Orthogonal Partial Least Squares Discrimination Analysis (OPLS-DA) was performed to determine metabolite features between groups (VIP > 1, *p* < 0.05). Other data were analyzed using GraphPad statistical software. The Shapiro–Wilk test was performed on the measures first, and then the unpaired t-test (normal distribution) or Kruskal-Wallis test (non-normal distribution) was used to compare the differences between the groups. Values of p < 0.05 were considered to be statistically significant. Finally, the statistical power (1-*β*) of the experimental design was calculated using the G*Power software, with a one-way ANOVA: Fixed effects, omnibus test applied.

## 3 Results

### 3.1 Participants characteristics

According to the evaluation criteria, eight healthy control children and five patients with ADHD were included. The individuals in both groups were male thus excluding the influence of sex. Table 1 displays the metadata for the two groups. The mean age of the ADHD group was (9.1 ± 0.9) years, while the mean age of the HC group was (8.9 ± 0.8) years. There was no statistically significant difference in age between the two groups (*p* = 0.74), indicating that age did not influence the study’s findings. The Conners Scale, Weiss’s Functional Degree of Impairment Scale, and the SNAP-IV Teacher Rating Scale were used to diagnose the ADHD patients.

**Table 1:** Donor metadata.

Donor ID	Group	Sex	Age	Diagnosis
HC-1	HC	M	9	NA
HC-2	HC	M	9	NA
HC-3	HC	M	8	NA
HC-4	HC	M	8.5	NA
HC-5	HC	M	9	NA
HC-6	HC	M	10	NA
HC-7	HC	M	8	NA
HC-8	HC	M	10	NA
ADHD-1	ADHD	M	9.9	ADHD
ADHD-2	ADHD	M	8.4	ADHD
ADHD-3	ADHD	M	8.9	ADHD
ADHD-4	ADHD	M	8.4	ADHD
ADHD-5	ADHD	M	10	ADHD

M, male; NA, not diagnosed.

### 3.2 Differences in microbiota composition between ADHD and healthy controls

**Figure 2:**
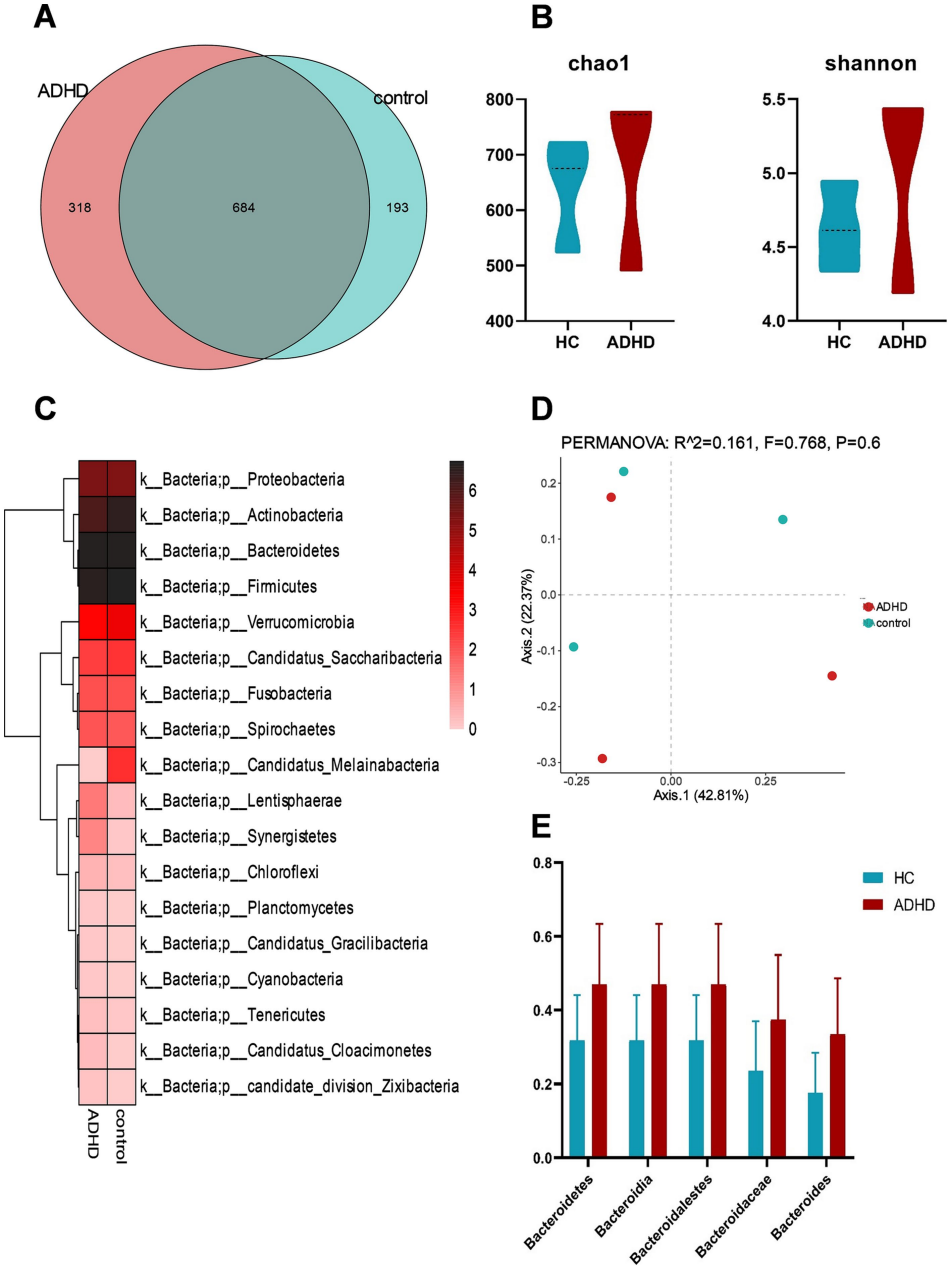
Analysis of bacterial diversity and composition. **(A)** Comparison of bacterial counts between the two groups; **(B)** Estimation of α-diversity using the Shannon and Chao1 indices (Shannon, *p* = 0.51; Chao1, *p* = 0.51); **(C)** Comparison of bacterial phyla levels between the two groups, with the highest relative abundances at the phylum level in both groups observed for *Bacteroidetes*, *Firmicutes*, *Actinobacteria*, and *Proteobacteria*; **(D)** PCoA analysis of β-diversity based on Bray-Curtis distance; **(E)** Comparison of relative abundances of Bacteroidetes phylum, class, order, family, and genus between the two groups, with all comparisons showing *p* > 0.05.

All samples underwent shotgun metagenomic sequencing. Nine distinct species types were identified in the two sets of samples, with bacteria accounting for the largest share at 87.16%. In total, 1,195 different bacterial species were found in the two sample groups; 318 of these species were unique to the ADHD group, while 193 species were unique to the HC group as shown in Figure 2A. There was no discernible difference between the ADHD and HC groups in terms of the bacterial groups’ alpha and beta diversity (Figure 2B). Examination of the sample composition showed that Bacteroidetes, Firmicutes, Actinobacteria, and Proteobacteria had the largest relative abundances in both groups at the phylum level (Figure 2C). As shown in Figure 2E, the relative abundances of *Bacteroidetes*, *Bacteroidia*, *Bacteroidalestes*, *Bacteroidaceae*, and *Bacteroides* were higher in the ADHD group than in the HC group, but no significant difference was observed (all *p* > 0.05).

**Figure 3:**
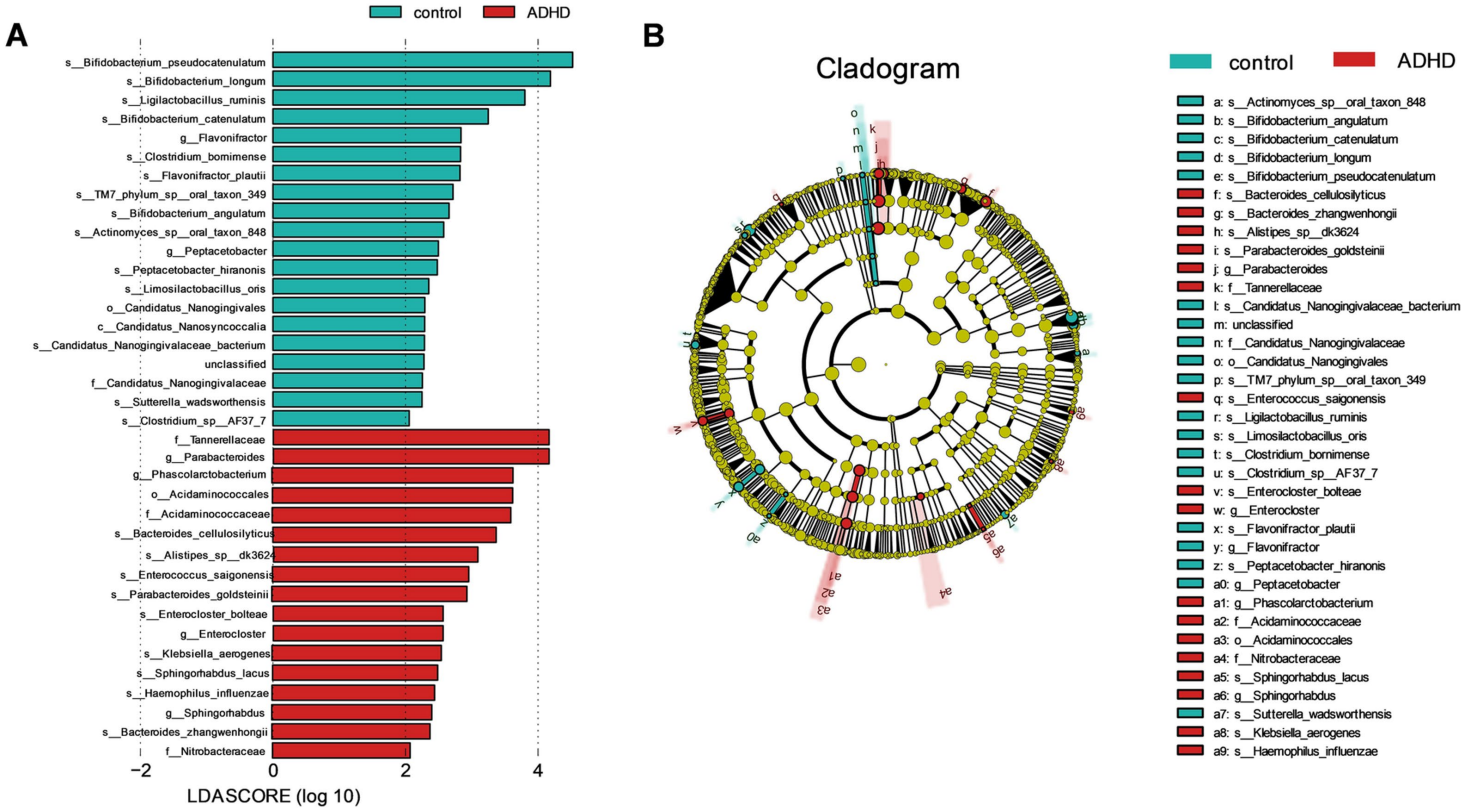
Significantly different bacteria. **(A)** Histogram of LEfSe analysis showing significantly different bacteria between the groups, categorized by group; **(B)** Evolutionary branching diagram of significantly different bacteria between the groups, sorted by relative abundance. *B. cellulosilyticus* exhibited the highest relative abundance in the ADHD group.

LEfSe analysis can be used to identify biological markers with substantial variations between subgroups. The LEfSe analysis results indicated that a total of 5 families, 7 genera, and 23 species had different relative abundances between the two groups (LDA score > 2.0, *p* < 0.05), as indicated in Table 2. Detailed results of all differential bacteria with *p* values are provided in Supplementary Table S1. Compared to the HC group, the ADHD group had a greater relative abundance of nine species, such as *Bacteroides cellulosilyticus* and *Enterococcus saigonensis*. Furthermore, the ADHD group had 61 characteristic KO genes. ADHD-specific bacterial species are shown in Figure 3. Column lengths represent the Linear discriminant analysis (LDA) values; the larger the LDA value, the larger the difference.

**Table 2:** Overview of significant microbiota.

	Decrease in ADHD	Increase in ADHD
Family level	Candidatus Nanogingivalaceae	Tannerellaceae Acidaminococcaceae Rhizobiaceae Nitrobacteraceae
Genus level	Peptacetobacter Flavonifractor	Parabacteroides Sphingorhabdus Phascolarctobacterium Bradyrhizobium Enterocloster
Species level	Bifidobacterium_pseudocatenulatum Bifidobacterium_longum Ligilactobacillus_ruminis Bifidobacterium_catenulatum Clostridium_bornimense Flavonifractor_plautii TM7_phylum_sp__oral_taxon_349 Bifidobacterium_angulatum Actinomyces_sp__oral_taxon_848 Peptacetobacter_hiranonis Limosilactobacillus_oris Candidatus_Nanogingivalaceae_bacterium Sutterella_wadsworthensis Clostridium_sp__AF37_7	Bacteroides_cellulosilyticus Alistipes_sp__dk3624 Enterococcus_saigonensis Parabacteroides_goldsteinii Enterocloster_bolteae Klebsiella_aerogenes Sphingorhabdus_lacus Haemophilus_influenzae Bacteroides_zhangwenhongii

### 3.3 Higher activity and curiosity were observed in SD rats receiving microbiota colonization from ADHD patients

**Figure 4:**
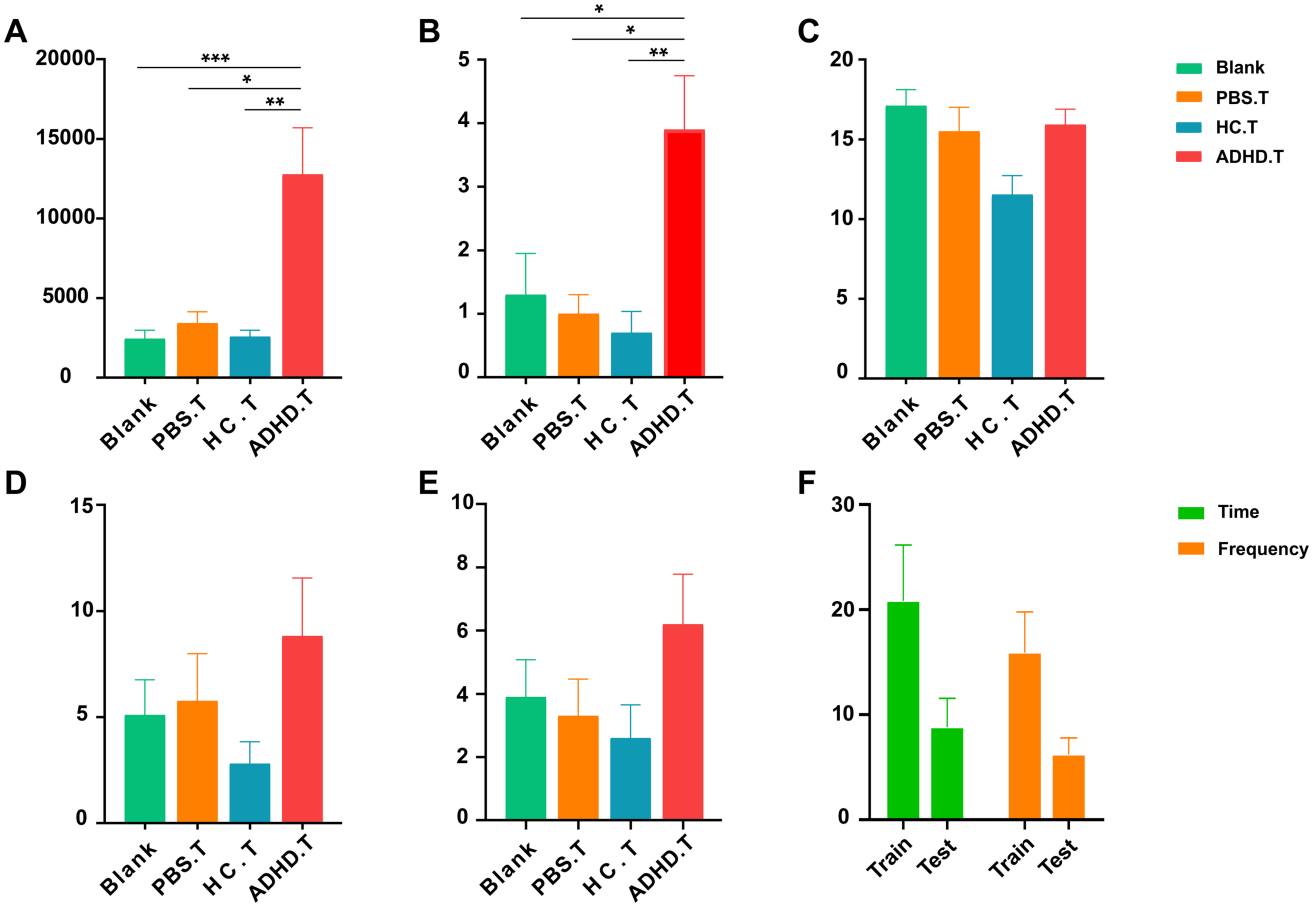
Behavioral results. **(A)** The total distance traveled in the OFT among the four groups. Significant differences were observed between the three control groups and the ratADHD group (Blank vs. ADHD.T: *p* = 0.0008; PBS.T vs. ADHD.T: *p* = 0.0333; HC.T vs. ADHD.T: *p* = 0.0037). **(B)** The number of times the animals stood upright in the OFT among the four groups. Significant differences were found between the three control groups and the ratADHD group (Blank vs. ADHD.T: *p* = 0.0197; PBS.T vs. ADHD.T: *p* = 0.0379; HC.T vs. ADHD.T: *p* = 0.0050). **(C)** Comparison of the number of marbles buried among the four groups of animals in the MBT. **(D)** Time spent exploring objects among the four groups of animals in the NOR. **(E)** Number of explorations of objects among the four groups of animals in the NOR. **(F)** Comparison between the training and testing phases of the NOR in the ADHD.T group. Blank: blank control group; HC.T, Health Control colony transplantation group; ADHD.T, ADHD colony transplantation group; PBS.T, sham operation group. *: *p* < 0.05; **: *p* < 0.01; ***: *p* < 0.001.

It is unknown how alterations in the microbiota of ADHD patients relate to symptoms of the disease, even though shotgun metagenomic sequencing data have shown that the gut microbiota of ADHD patients differed from that of healthy persons. In order to investigate this relationship further, we conducted several behavioral tests on SD rats after isolating and transplanting the gut microbiota from ADHD patients and HCs. Furthermore, two sham-operated groups were established: one with PBS intervention and the other blank control group receiving no treatment at all. In contrast to the other three groups, the OFT results demonstrated a significant increase in the rat^ADHD^ group’s total distance traveled in terms of activity and the number of uprights in the open field. This suggested that the rat^ADHD^ group exhibited higher levels of movement and a stronger interest in external exploration, which is consistent with behavior that is similar to ADHD. The other measures in the OFT, such as the number of entries into the central zone, the distance traveled, and the amount of time spent in the central zone did not differ substantially across the groups, as depicted in Figure 4.

In the training portion of the NOR experiment, which evaluates memory, all rats were equally able to investigate two similar objects. The rat^ADHD^ group did not exhibit a trend to lower the discrimination index when compared to the control groups throughout the 24-h test phase. However, they did investigate new objects less frequency and fewer times overall than they did during the training phase. The MBT can be used to identify obsessive or impulsive behaviors in animals. Our research revealed no discernible variation in the number of marbles buried in the rat^ADHD^ group when compared to the other rat groups. All data and analyses from the behavioral experiments are presented in Supplementary Tables S2, S3.

To evaluate the validity of the experimental design and its ability to detect group differences, a statistical power analysis was performed on the key behavioral indicators mentioned above. The results presented in Table 3 were obtained with a significance level of *α* = 0.05, a total sample size of 40, and a group size of four. The analysis revealed that the test power for the total distance traveled in the OFT and the number of uprights in the OFT was high, indicating a reliable capacity to detect group differences. In contrast, the test power for the NOR experiment and the number of burials in the MBT was low, suggesting a risk of insufficient power to detect meaningful differences.

**Table 3:** Statistical power of behavioral experimental indicators.

	SD within each group	Effect size f	Power (1-β err prob)
OFT distance	4871.84	0.888	0.997
OFT uprights	1.83	0.696	0.952
NOR time	6.39	0.336	0.363
NOR trials	3.99	0.339	0.368
MBT burials	4.11	0.437	0.581

### 3.4 Metabolite nicotinamide may be correlated with ADHD

**Figure 5:**
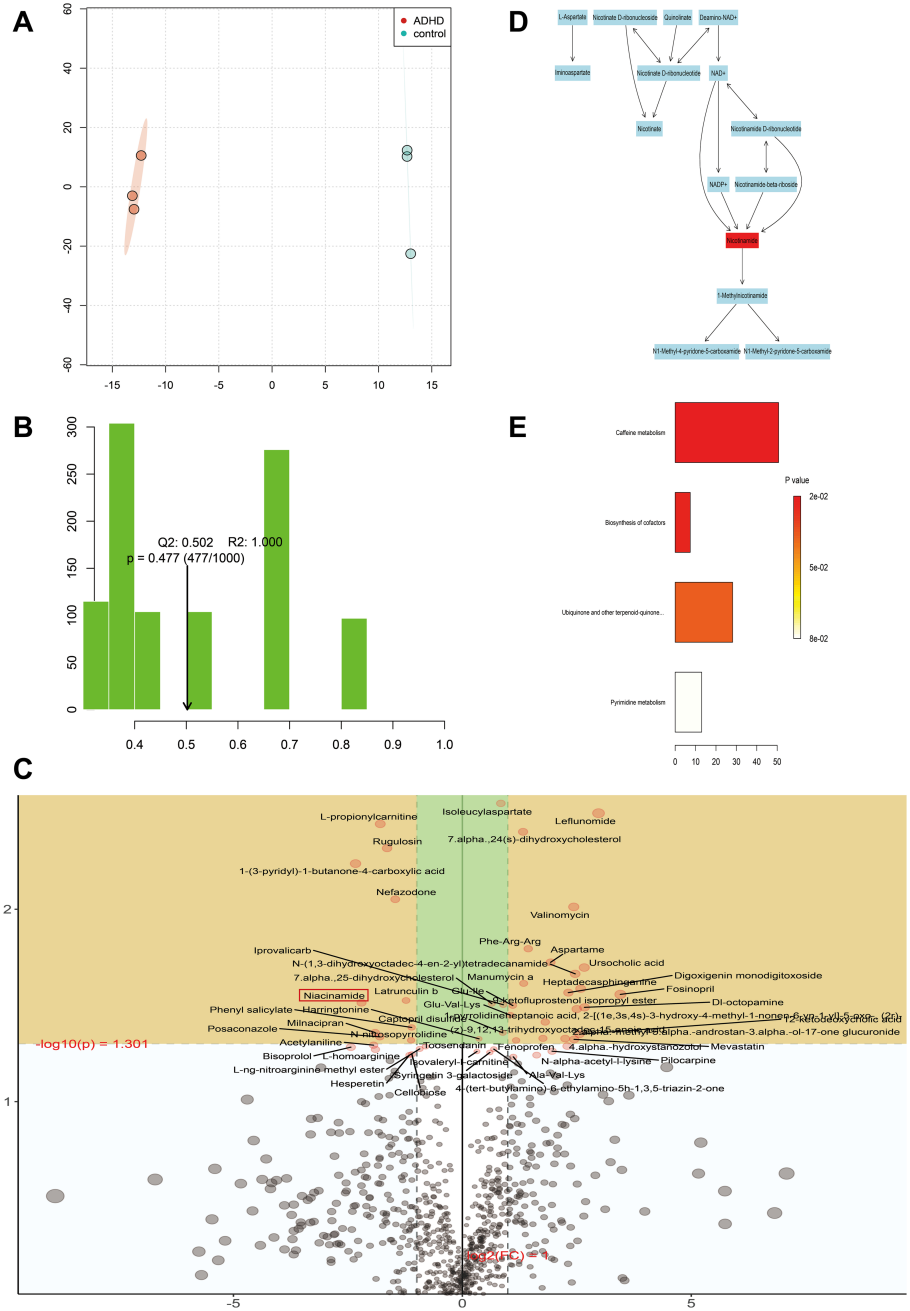
Metabolite analysis. **(A)** Metabolite cluster separation between the two groups is clearly defined. The horizontal axis represents differences between groups, while the vertical axis represents differences within samples of each group. **(B)** The *R*^2^ and *Q*^2^ values represent the variance explained by the model and the predictability of the model, respectively. Our model demonstrates high reliability and strong predictive ability. **(C)** Each point represents a specific metabolite. Metabolites located in the yellow area are significantly differentially expressed between the two groups. Points in the upper left indicate metabolites with downregulated relative differential expression in the ADHD group, while points in the upper right indicate metabolites with upregulated relative differential expression in the ADHD group. The closer a point is to the left, right, or top edges of the graph, the more significant the difference in expression. **(D)** Schematic of the reaction network for nicotinamide-related metabolites. Red markers highlight nicotinamide as a key metabolite requiring special attention. **(E)** Negative ion mode ORA enrichment results suggest that these metabolic pathways may also warrant further focus.

Metabolites in feces were further analyzed to assess whether there were differences between ADHD and HC metabolite groups. The characteristics that differed most between subgroups were chosen using OPLS-DA. The association between metabolites expression and grouping was modeled by OPLS-DA, providing improved access to intergroup differences and enabling prediction of sample grouping, which could not be accomplished using PCA. The ADHD and HC metabolite clusters were clearly distinguished as shown in Figure 5A, where the horizontal coordinates show differences between groups, and the vertical coordinates show differences between samples within groups. The positive ion model plot is depicted in Figure 5B; *R*^2^ and *Q*^2^ represent the variables that the model explained and the degree of predictability, respectively. These values allow for the differentiation of the model’s advantages and disadvantages. Figure 5B illustrates that the positive ion model accepted both *R*^2^ and *Q*^2^. Eight hundred fifty-six positive ion metabolites were found in the positive ion model; of these, 26 were considerably up-regulated (*p* < 0.05, VIP > 1) in ADHD compared to HCs, and 10 were significantly down-regulated (*p* < 0.05, VIP > 1), as shown in Figure 5C.

**Figure 6:**
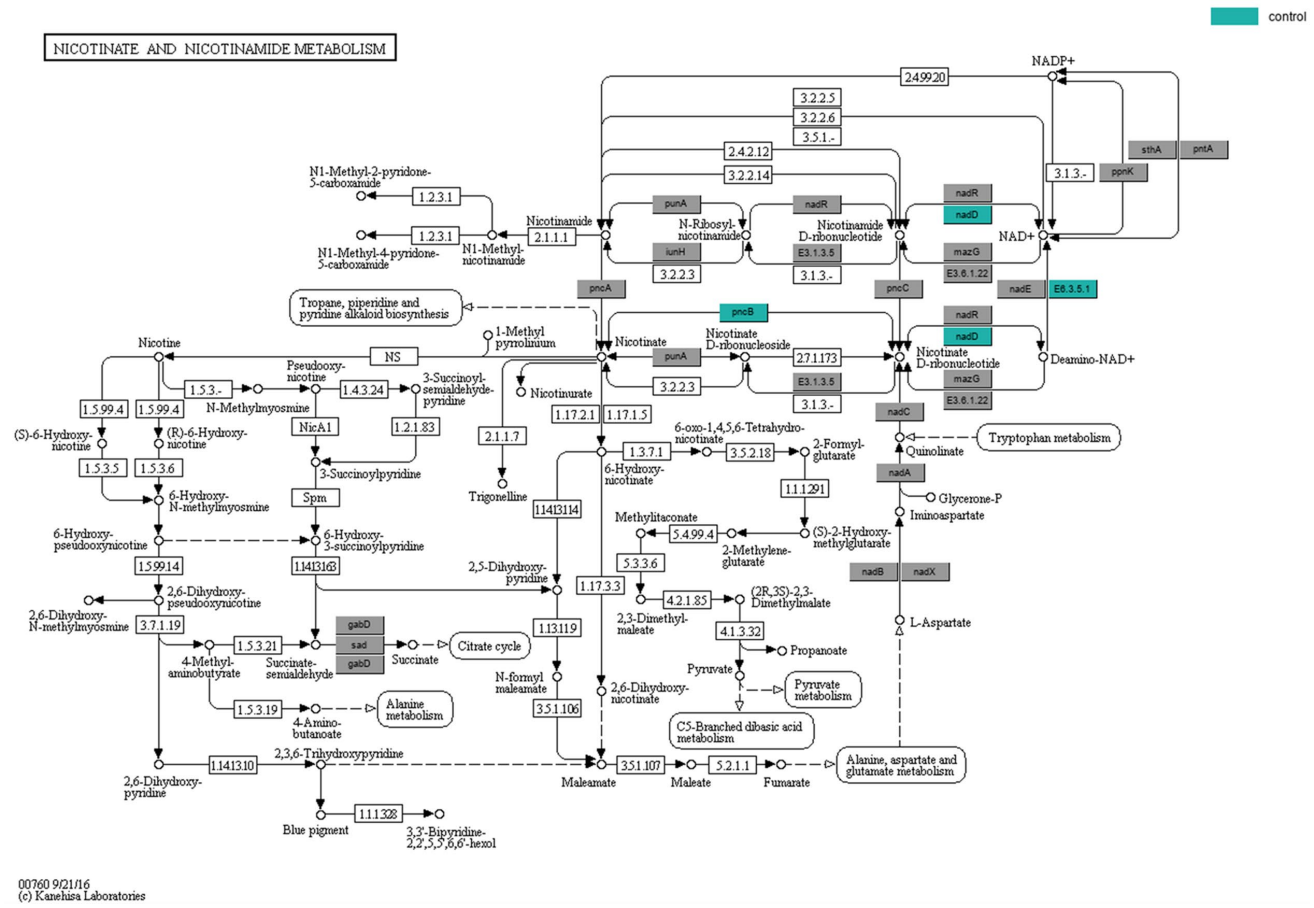
Nicotinic acid and nicotinamide metabolic pathway. The color-coded genes, nadD and pncB, represent critical sites in the metabolic pathway.

KEGG enrichment analysis and pathway annotation of metabolites from both groups were used to further examine the metabolic effects of ADHD. The Oracle database was utilized to identify the KEGG pathways that were significantly enriched for the particular set of metabolites that were obtained from OPLS-DA analysis. The topological influence was then computed, which served as an anchor for the significantly enriched KEGG pathways (Figures 5D, E). The findings demonstrated some metabolic pathways such as nicotinamide and nicotinic acid metabolism in the positive ionic pattern, as well as the four negative ionic metabolic pathways: cofactor biosynthesis, ubiquinone and other terpene quinone biosynthesis, pyrimidine metabolism, and caffeine metabolism. It was possible that the development of disease was correlated with certain metabolic pathways. When the findings of the metagenomic sequencing analysis of colony function were integrated, three distinct genes were identified, namely *pncB* (K00763), *nadD* (K00969), and *E6.3.5.1* (K01950), that exhibited comparatively lower expression in the ADHD group in the nicotinic acid and nicotinamide metabolic pathways. The expression of the positive ionic metabolite nicotinamide was substantially down-regulated in the ADHD group compared to the HC group (*p* = 0.03, VIP = 1.87), which is consistent with the findings of untargeted metabolomics (Figure 6).

## 4 Discussion

ADHD is a common neurodevelopmental disorder. While many studies have shown that the gut microbiota of patients with ADHD differed from that of healthy individuals, it is still unclear exactly how gut flora contribute to the pathophysiology of ADHD, and it is impossible to identify the specific changes that occur in the gut flora of ADHD patients based on previous research. The majority of research has employed 16S RNA sequencing to detect variations in the gut microbiome between healthy and ADHD patient groups; however, intergroup differences in gut flora cannot be examined at the species level using 16S sequencing. This study focused on the species-level composition and abundance of bacteria and examined the functional differences between them by applying shotgun metagenomic sequencing to investigate the structural and compositional features of the gut flora in both healthy populations and ADHD patients. All clinical samples in this study were collected from male patients, as previous literature indicates significantly higher rates of pediatric ADHD diagnoses in males compared to females (Faraone et al., 2015; Reuben and Elgaddal, 2024). Notably, the male-to-female diagnosis ratio in epidemiological studies has risen from 3–4:1 to 7–8:1, underscoring the predominance of males in ADHD diagnoses (Thapar and Cooper, 2016). Consequently, our findings may primarily reflect the characteristics of ADHD in male patients. According to the shotgun metagenomic sequencing data, there was no discernible change in *α*-diversity and *β*-diversity between the two sample groups, which is consistent with some earlier research findings (Jiang et al., 2018; Wan et al., 2020; Wang et al., 2022, 2023). However, other research has demonstrated notable distinctions between ADHD patients and healthy individuals in terms of gut microbiota α-diversity (Jung et al., 2022) or β-diversity (Szopinska-Tokov et al., 2020). Geographical location, eating practices, sample selection, and other environmental factors could all contribute to these variations. For instance, dietary intake of dairy products, nuts, and legumes in ADHD patients showed a significant association with *S. stercoricanis* (Wang et al., 2020). The development of ADHD may include the gut microbiota in a complex way, and the main markers that set apart children with ADHD from healthy children may not be α-diversity or β-diversity.

Bacteroidetes, Firmicutes, Actinobacteria, and Proteobacteria were the phylum-level bacteria with the highest relative abundance in both groups, according to our data. As the two main bacterial groups participating in the pathways leading to the metabolism of sugar and transporters, respectively, Firmicutes and Bacteroidetes are known to exist (Meehan and Beiko, 2012). The ratio of Firmicutes to Bacteroidetes, or F/B, is a crucial indicator for measuring the extent of gut microbiota disorder. The abundance of Bacteroidetes to Bacteroides in ADHD was higher than that in HCs, according to the results of sample composition analysis at each biological level. This suggested that F/B was trending downward in ADHD, indicating that the gut microbiota in ADHD was different to that in HCs, albeit not significantly. Based on the intergroup LEfSe study, the ADHD group had a higher relative abundance of *Bacteroides cellulosilyticus* than the HC group, and there were changes in bacterial abundance in three orders, five families, seven genera, and 23 species. Several studies have shown that patients with inattentive-ADHD had a higher relative abundance of *B. cellulosilyticus* when compared to HCs, consistent with the results of our study (Shirvani-Rad et al., 2022; Tas and Ulgen, 2023). Additionally, one report indicated that this bacterium was negatively correlated with symptoms of hyperactivity/impulsivity (DSM_HD scores) and attention deficit (DSM_AD scores) in ADHD patients (*p* < 0.001; Li et al., 2022), implying that *B. cellulosilyticus* may play a beneficial role in the gut microbiota of patients with ADHD. Imbalances in the gut microbiota are closely associated with the onset and progression of ADHD. Gut microbes can influence cognitive function through short-chain fatty acids (SCFAs) or tryptophan metabolism (Desbonnet et al., 2008; Selkrig et al., 2014). As one of the major metabolites of gut microbiota, SCFAs regulate host metabolism through multiple pathways, including regulating glucose and lipid metabolism, maintaining the intestinal barrier, and mitigating inflammatory responses (Koh et al., 2016). Plasma concentrations of SCFAs have been observed to decrease in ADHD patients (Yang et al., 2022), and Bacteroides is among the known producers of SCFAs (Koh et al., 2016). Therefore, we hypothesize that intermediate metabolites like SCFAs may serve as key breakthroughs in elucidating the link between behavioral alterations in ADHD and Bacteroides. The relative abundance of *B. cellulosilyticus* was found to be down-regulated in obese populations that were metabolically healthy (Chen et al., 2022) and in individuals who had atherosclerosis (Jie et al., 2023). The enrichment of *B. cellulosilyticus* was linked to healthy dietary practices that decreased the risk of cardiometabolic illnesses. This correlation may stem from the polysaccharide utilization loci (PUL) function of *B. cellulosilyticus*. The PULs in Bacteroides species are thought to include secreted glycosidases, uptake transporter proteins embedded in the cytoplasmic membrane, and cytoplasmic enzymes involved in carbohydrate metabolism (Zafar and Saier, 2021). These intricate PUL systems enable many Bacteroides species to initiate and carry out the utilization and metabolism of carbohydrates. Therefore *B. cellulosilyticus* may be beneficial in preventing or treating obesity and disorders associated with obesity. Furthermore, it is believed that *B. cellulosilyticus* results in diminished anti-inflammatory actions because some Bacteroides species may participate in intestinal immune responses through mechanisms such as protein mimicry, protein export and regulation of surface polysaccharides (Arnolds et al., 2023; Wang et al., 2021). None of the currently identified changes in bacterial taxa fully explain their precise relationship with ADHD symptoms, highlighting an area that warrants further investigation in future research.

We aimed to further verify whether there was a connection between gut flora and ADHD-like behaviors based on the findings of shotgun metagenomic sequencing analysis that could detect changes in the gut microbiota between ADHD patients and HCs. FMT is frequently employed to confirm the existence and development of gut flora in a range of illnesses (Antushevich, 2020). Thus, this study used FMT to confirm the link between gut flora and ADHD behaviors. After transplanting fecal flora from both the HC population and ADHD patients into SD rats, we simultaneously established two control groups and conducted a series of behavioral experiments to determine whether the rats that received colonization displayed distinct behavioral responses. According to the findings, SD rats colonized with ADHD gut microbiota exhibited higher locomotor activity and curiosity than the other three groups, which is consistent with behaviors similar to ADHD. This suggests that ADHD flora may contribute to the development of behaviors similar to ADHD. As other results (Tengeler et al., 2020) do not agree with ours, it was theorized that this might be because of the vast variations in animal individuality and the many transplantation techniques. To evaluate the validity of the behavioral experimental design and the reliability of the data, this study reported high test efficacy for the OFT total distance traveled and number of uprights through statistical efficacy analyses. These results highlight the significance of between-group differences and the efficiency of the experimental design. This indicates that in experiments investigating animal activity and curiosity, the current sample size and design were sufficient to effectively capture treatment effects and ensure both reliability and statistical significance. The relatively lower test efficacy observed in other experiments may stem from individual animal differences and the varying sensitivity of behavioral indicators. Compared to the OFT, which directly reflects the animal’s state, NOR and MBT involve more complex cognitive and behavioral patterns that may require larger sample sizes or more refined experimental conditions to minimize data variability. Nonetheless, despite the lower test efficacy, the between-group means for exploration time and number of explorations in NOR exhibited observable trends, offering valuable directions for further investigation into related behavioral mechanisms.

Researching the relationship between gut microorganisms and disease alone is insufficient as the gut contains a large number of metabolites, most of which require the assistance of the gut flora for production and breakdown. Consequently, we carried out an untargeted metabolomics investigation. By combining OPLS-DA analysis and KEGG enrichment analysis, the metabolism of nicotinamide and niacin was found to be enriched in the positive ion mode. The metabolite nicotinamide was the highlight of this analysis. According to our study findings, ADHD patients had lower nicotinamide levels than HCs. Nicotinamide, a form of vitamin B3, has been linked to the growth, survival, and function of neurons in the central nervous system and may be beneficial in the prevention and improvement of neurocognitive function (Rennie et al., 2015), as well as in conditions such as depression, ischemia, stroke, and schizophrenia (Fricker et al., 2018; Song et al., 2019). Monoamine neurotransmitter levels are maintained in cells by nicotinamide (Song et al., 2019). Nicotinamide is a precursor of nicotinamide adenine dinucleotide (NAD+), and the level of NAD+ may be involved in one of the possible processes of nicotinamide’s actions. NAD+ plays critical roles in DNA repair, cell signaling, and cellular energy metabolism (Dhuguru et al., 2023; Niño-Narvión et al., 2023). Its concentration particularly impacts nerve cell survival and function in the neurological system. Dysfunction of neural cells resulting from abnormal NAD+ metabolism may influence cognitive and behavioral processes (Rennie et al., 2015). The health of neurons may be significantly impacted by the harmonious interaction between nicotinamide and NAD+.

Reduced nicotinamide levels in ADHD may indicate a problem with NAD+ metabolism. Disproportions in NAD+ metabolism could be linked to symptoms of ADHD, given the significance of NAD+ in neurological function. For instance, imbalances in NAD+ metabolism may impact neurotransmitter synthesis and release, nerve cell energy metabolism and communication (Chellappa et al., 2022; Shats et al., 2020). NAD+ metabolism is regulated by both nicotinate-nucleotide adenylyltransferase (*nadD*) and nicotinate phosphoribosyltransferase (*pncB*), the two characteristic genes whose relative expression was found to be decreased in ADHD in the functional analysis of macrosequencing. Overexpression of the *pncB* gene in a strain resulted in an increase in NAD+ production (Wu et al., 2021). *NadD* is a crucial enzyme in the NAD+ metabolic pathway (Ajunwa et al., 2022; Rodionova et al., 2015). Modulating the NAD+ metabolic pathway may become a viable therapeutic strategy if the anomalies of nicotinamide and NAD+ metabolism in ADHD can be further proven. Increasing NAD+ production by supplementing with nicotinamide may help reduce some of the symptoms associated with ADHD. Furthermore, one study (Mallya et al., 2023) that examined the use of dietary supplements and herbs to treat ADHD noted that dietary supplements are helpful for the disorder and that nicotinamide may be a useful supplement. Additionally, nicotinamide has been used to treat behavioral disorders, migraine headaches, and depression (Tian et al., 2013).

In conclusion, our study provided a comprehensive list of the metabolites and fecal bacteria that were changed in ADHD, especially the species-level microbes found. Our study’s strength was that, in comparison to single-omics studies, the use of a combined multi-omics analysis provided a fuller and complementary understanding of ADHD, assisting researchers in better understanding the condition. Further research is necessary to ascertain the possible influence of gut microbiota and metabolites on ADHD, given the advancement of gut microecological assays. This study has several limitations that should be acknowledged. The clinical sample size was relatively small and the geographic selection area was restricted, which may limit the diversity of the participant population. Additionally, the imbalance in the male-to-female ratio could have influenced the observed differences in the intestinal microbiota. As the findings predominantly reflect the characteristics of ADHD in male patients, future studies should strive to include a more balanced representation of genders to enhance the generalizability and robustness of the results. Furthermore, to validate and expand upon our findings, it is essential to conduct larger-scale and more diverse studies. Such efforts could help overcome current limitations by investigating factors such as environmental and lifestyle influences. Overall, this multi-omics study methodically examined the possible relationship between alterations in gut flora, fecal metabolites and their enrichment pathways with ADHD. This study has the potential to offer an experimental foundation for future investigations into the functions and impacts of gut microbiota and metabolites in ADHD.

## Data Availability

The original contributions presented in the study are publicly available. This data can be found at: https://www.ncbi.nlm.nih.gov/sra, BioProject ID PRJNA1222598. The associated BioSample IDs are as follows: SAMN46785179, SAMN46785180, SAMN46785181, SAMN46785182, SAMN46785183, and SAMN46785184.
