# Erratum: Integrative analysis of intestinal flora and untargeted metabolomics in attention-deficit/hyperactivity disorder

**DOI:** 10.3389/fmicb.2025.1576969

**Published:** 2025-02-27

**Authors:** Frontiers Production Office

**Affiliations:** Frontiers Media SA, Lausanne, Switzerland

**Keywords:** attention deficit hyperactivity disorder, intestinal flora, metagenomics, metabolomics, fecal microbial transplantation

Due to a production error, the Data Availability Statement (DAS) was not correctly captured upon publication. The corrected DAS appears below:

“The original contributions presented in the study are publicly available. This data can be found at: https://www.ncbi.nlm.nih.gov/sra, BioProject ID PRJNA1222598. The associated BioSample IDs are as follows: SAMN46785179, SAMN46785180, SAMN46785181, SAMN46785182, SAMN46785183, and SAMN46785184.”

The publisher apologizes for this mistake.

The original version of this article has been updated.

