# Supplementary material for: Integrative analysis of intestinal flora and untargeted metabolomics in attention-deficit/hyperactivity disorder
Source: Front Microbiol. 2025 Jan 29;16:1452423. doi: 10.3389/fmicb.2025.1452423 (PMC11817268; doi:10.3389/fmicb.2025.1452423)

Supplementary figure 1 OFT distance


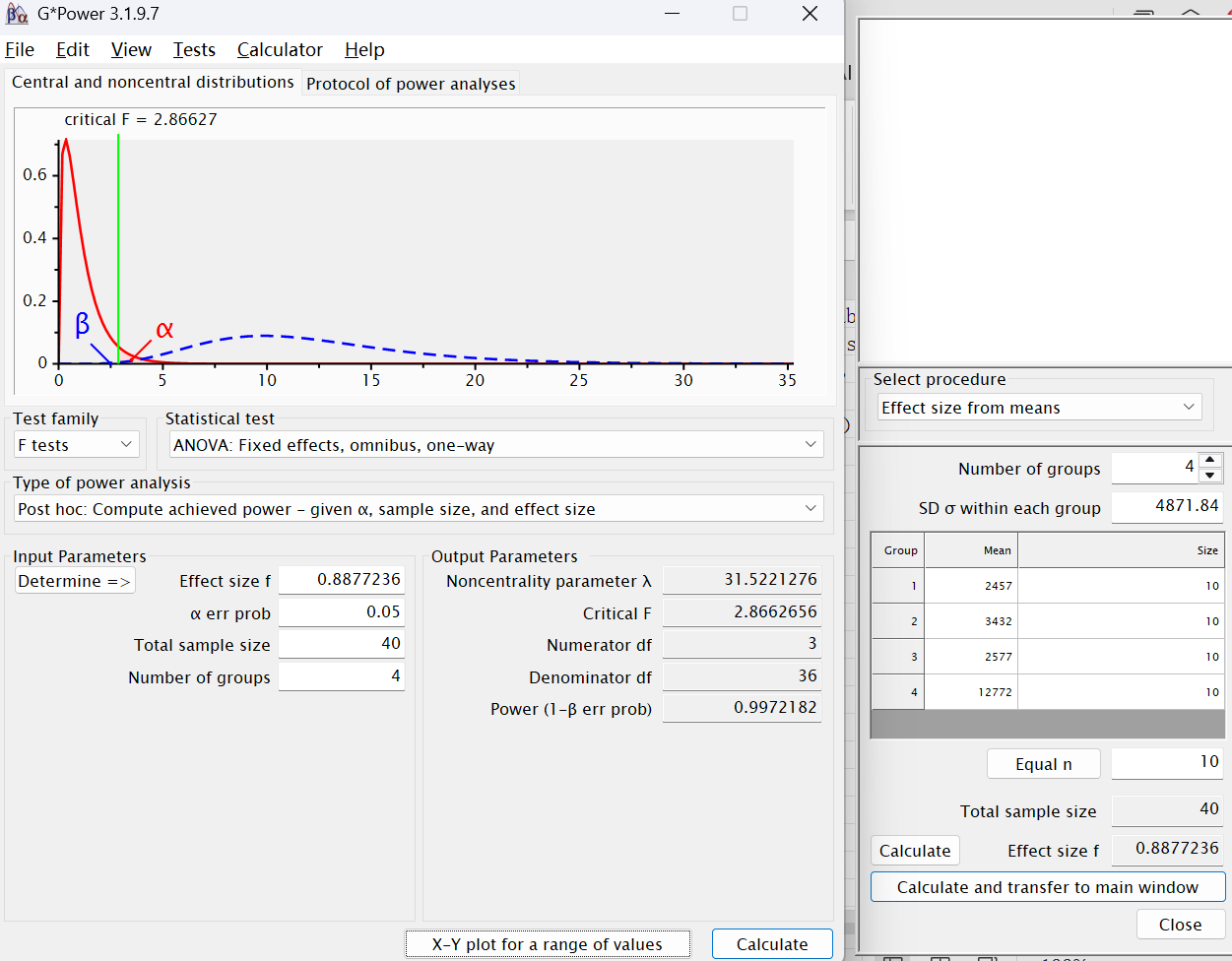


Supplementary figure 2 OFT uprights


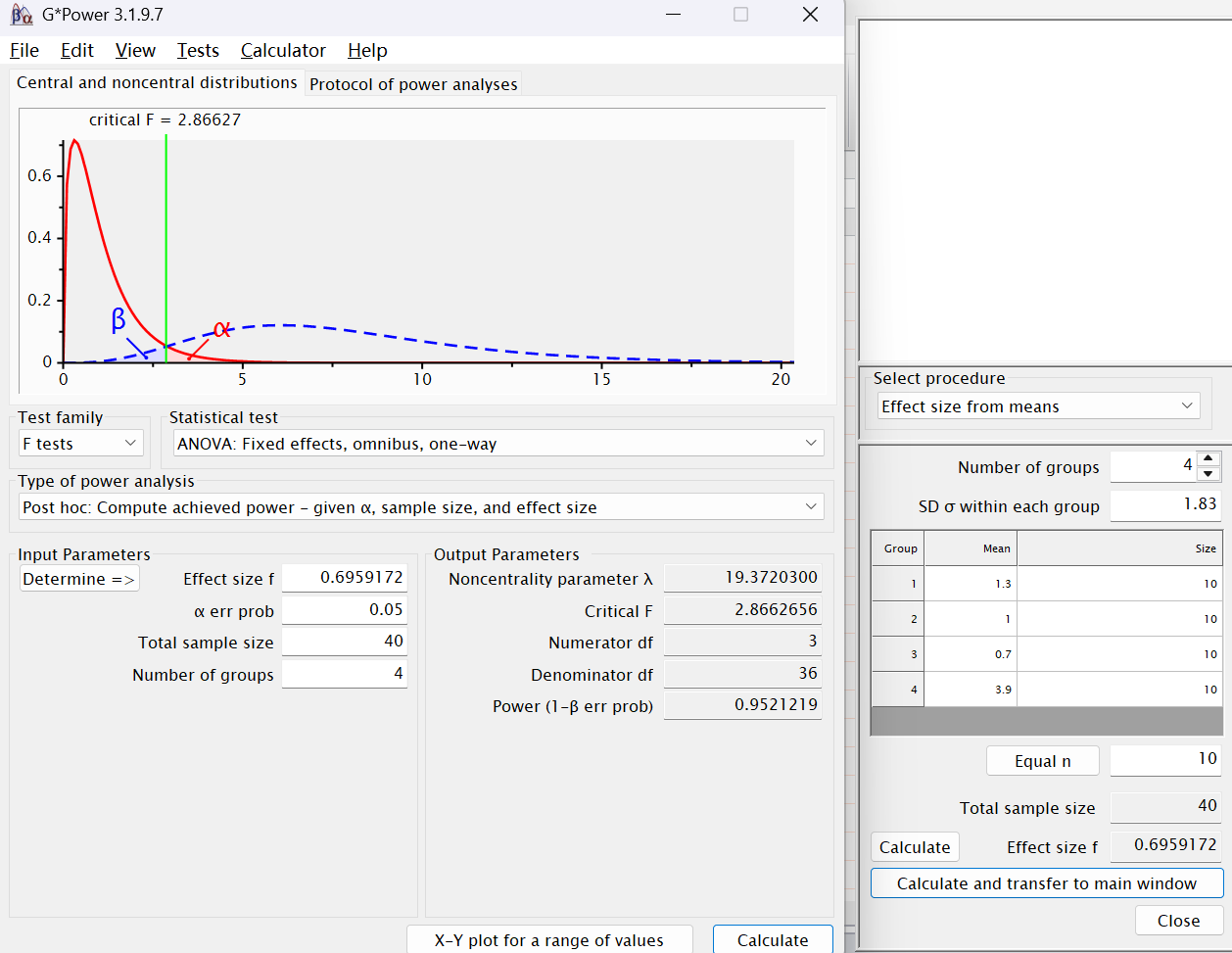


Supplementary figure 3 NOR time


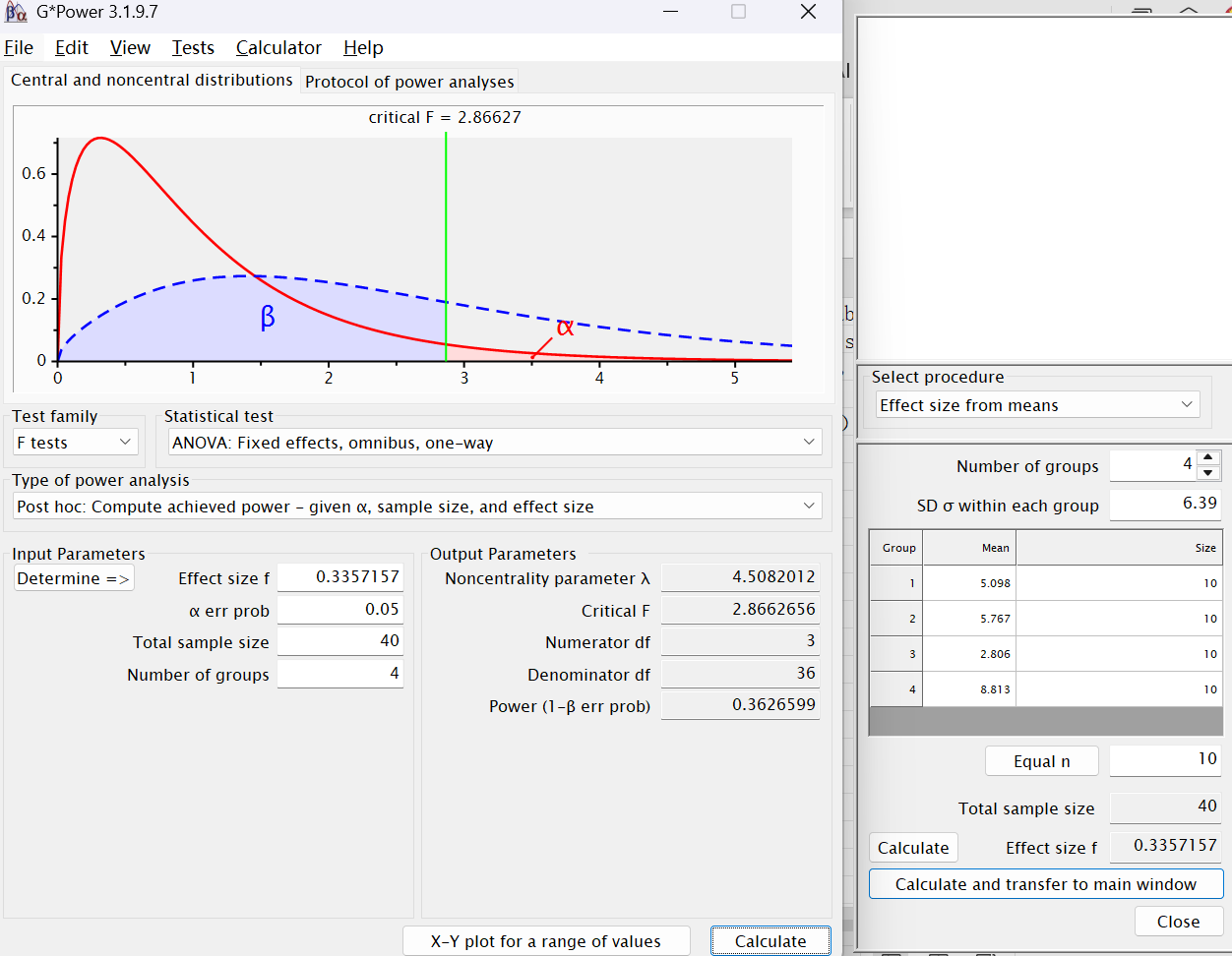


Supplementary figure 4 NOR trials


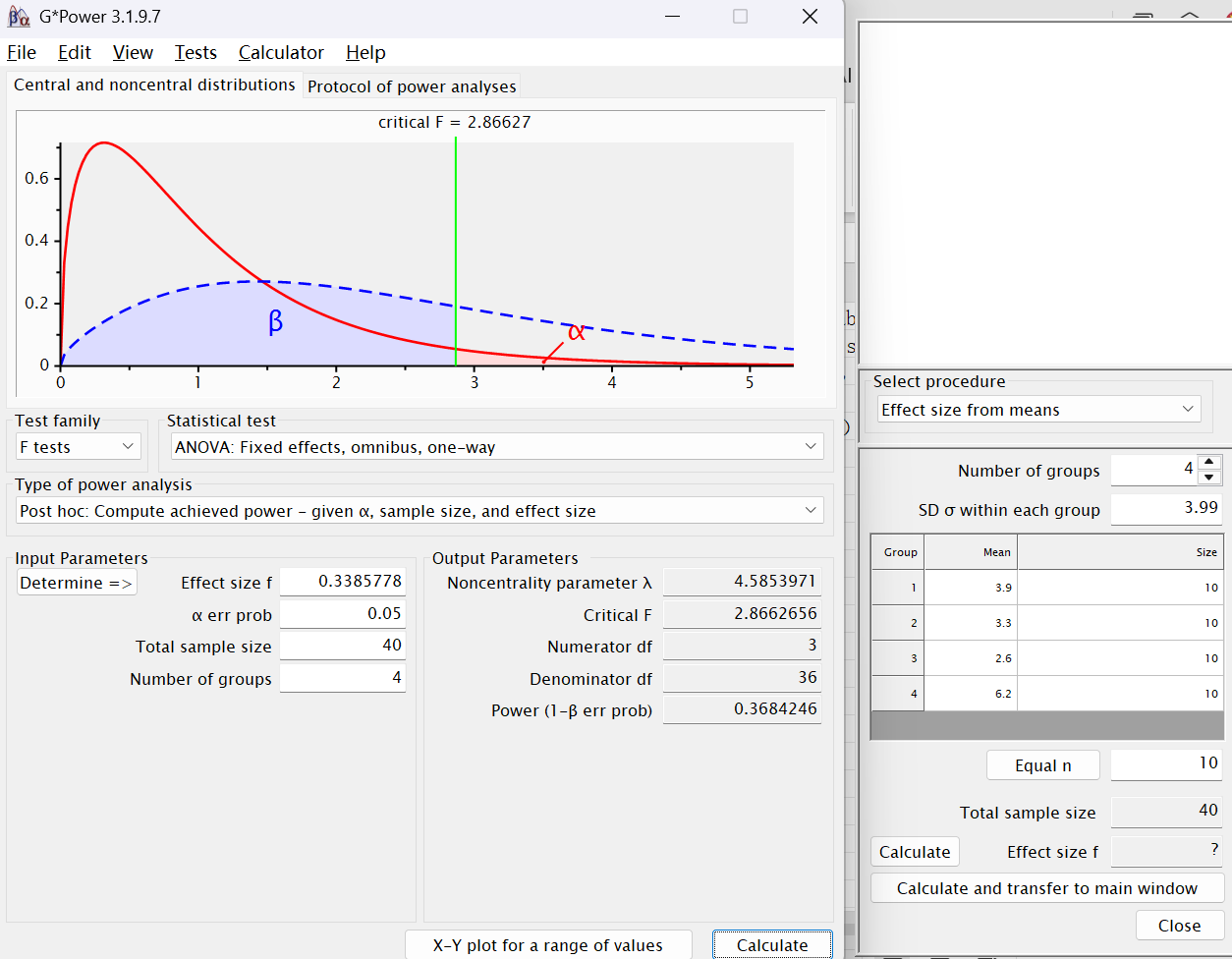


Supplementary figure 5 MBT burials


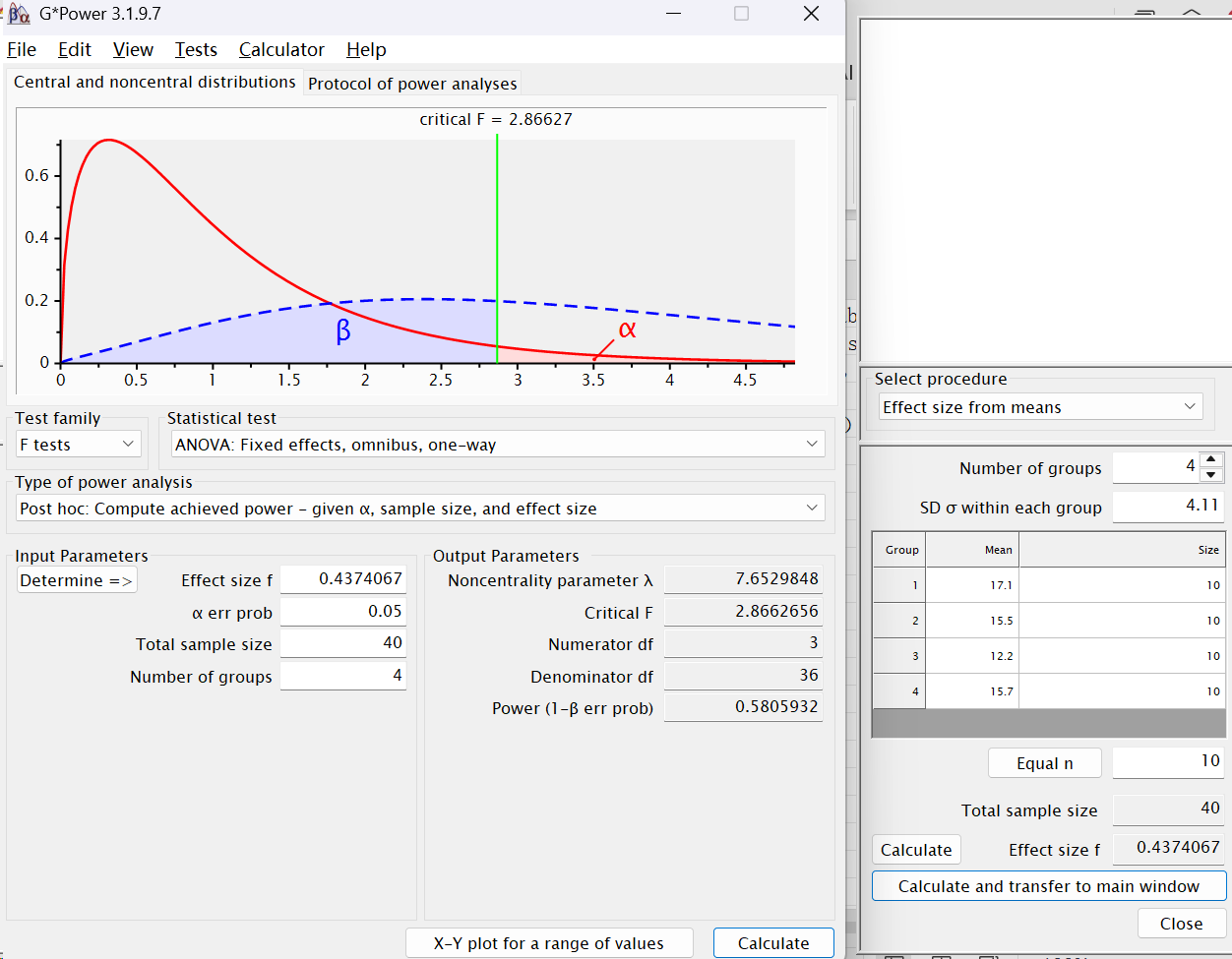

Supplement: Supplementary file 4 [file Data_Sheet_1.docx]
